# Emerging role of non-coding RNA in neural plasticity, cognitive function, and neuropsychiatric disorders

**DOI:** 10.3389/fgene.2012.00132

**Published:** 2012-07-13

**Authors:** Paola A. Spadaro, Timothy W. Bredy

**Affiliations:** Psychiatric Epigenomics Laboratory, Queensland Brain Institute, The University of Queensland,Brisbane, QLD, Australia

**Keywords:** non-coding RNA, neuropsychiatric disorder, addiction, anxiety

## Abstract

Non-coding RNAs (ncRNAs) have emerged as critical regulators of transcription, epigenetic processes, and gene silencing, which make them ideal candidates for insight into molecular evolution and a better understanding of the molecular pathways of neuropsychiatric disease. Here, we provide an overview of the current state of knowledge regarding various classes of ncRNAs and their role in neural plasticity and cognitive function, and highlight the potential contribution they may make to the development of a variety of neuropsychiatric disorders, including schizophrenia, addiction, and fear-related anxiety disorders.

## INTRODUCTION

*From whence we came, whither we go: the rise of the RNA world.* When Woese, Orgel, and Crick each wrote about the origin and evolution of the genetic code they laid the foundation for “The RNA world hypothesis,” which was later developed by Gilbert (Woese, 1967; Crick, 1968; Orgel, 1968; Gilbert, 1986). This hypothesis stemmed from the fact that ancient RNA had the ability to transmit heritable information as well as, in the case of ribozymes, being endowed with enzymatic capacity (Cech et al., 1981; Kruger et al., 1982; Guerrier-Takada et al., 1983; Joyce, 2002; Cech, 2011). With the advent of next-generation sequencing technology, coordinated efforts such as the ENCODE and FANTOM 3 projects have revealed that more than 90% of the genome is actively transcribed, whereas only ~2% codes for functional protein. These landmark observations brought into question the true value of RNA as a regulatory molecule and have since motivated scientists to embark on a deeper exploration of the biological function of so-called “junk DNA” or “genomic dark matter” (Johnson et al., 2005; Derrien et al., 2011). Many non-coding RNAs (ncRNAs) are highly conserved and correlate with the degree of eukaryotic complexity, further strengthening the argument that they are more than simple artifacts of evolution (Griffiths-Jones et al., 2005; Taft et al., 2007; Heimberg et al., 2008; Mattick, 2011). In fact, their ability to drive transcription, direct epigenetic processes, and silence gene expression make ncRNAs ideal candidates for insight into molecular evolution and a better understanding of the molecular pathways of neuropsychiatric disease. In this review, we provide an overview of the current state of knowledge regarding the involvement of various classes of ncRNAs in neural plasticity as well as their potential role in a variety of neuropsychiatric disorders, particularly those characterized by impairments in cognitive function.

## NON-CODING RNA: A KEY DRIVER OF GENE REGULATION

The broad potential of ncRNAs to critically regulate transcription and translation has been appreciated for some time with the discovery and characterization of various ncRNA families, the majority of which have been shown to be capable of directing epigenetic processes (Mattick and Makunin, 2006). A simplistic division of the different classes of ncRNAs can be made based on their size: small ncRNAs, between 20 and 200nt, and long ncRNAs, arbitrarily identified as any ncRNA over 200 nt. Small ncRNAs amazed the scientific world when they were identified as in *Caenorhabditis elegans* as being involved in the post-transcriptional regulation of genes during development (Lee et al., 1993; Wightman et al., 1993). Moreover, Fire and Mello were awarded a Nobel prize in 2006 for their discovery of RNA interference in *C. elegans* (Fire et al., 1998). Long ncRNAs have also recently entered the spotlight. For example, Rinn et al. (2007) recently discovered a long ncRNA called HOTAIR, which regulates epigenetic control over the HOXD locus, thereby providing novel evidence for a functionally relevant role for this enigmatic family of non-coding transcripts.

## SMALL ncRNAs: miRNAs

**FIGURE 1:**
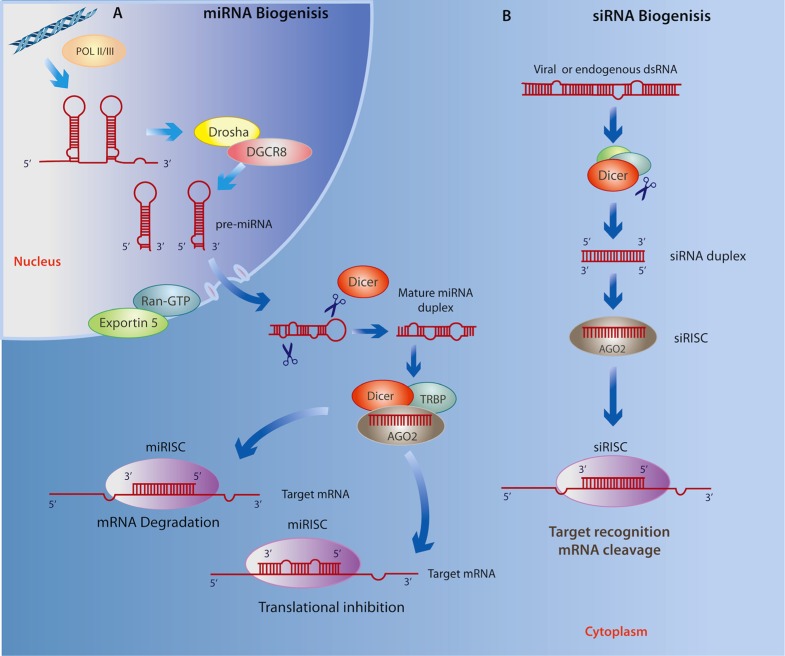
**(A)**miRNA biogenesis begins in the nucleus where *Drosha* and DGCR8 cleave the primary RNA resulting in a miRNA precursor that is exported to the cytoplasm by Exportin5 and Ran-GTP61 where it is then processed by *Dicer* and TRBP. Next, the mature miRNA duplex is incorporated to the RISC where it is cleaved *Dicer*, associates with AGO2, and subsequently binds to the 3′ UTR of a target RNA leading to either mRNA degradation or translational repression. **(B)** Similarly, siRNA biogenesis depends on *Dicer* activity and incorporation into the RISC, however siRNAs avoid cleavage by *Drosha* as they are derived from exogenous viral or endogenous double-stranded RNA molecules mostly within the cytoplasm. Given the specificity of base pairing, the final outcome of the siRNA interference is the cleavage and degradation of their target mRNAs.

MicroRNAs (miRNAs) are a class of endogenous, small ncRNAs that mediate post-transcriptional gene silencing by complementary binding to the 3′-untranslated region (3′ UTR) of their target mRNA, a key process for regulating gene expression in a tissue- and developmental stage-specific manner (Liu and Kohane, 2009). As illustrated in **Figure 1A**, the biogenesis of miRNAs begins in the nucleus through a canonical pathway as a large primary-RNA (pri-RNA) molecule, which folds into a stem loop transcribed by both RNA polymerase II (RNA Pol II) and RNA polymerase III (RNA Pol III; Kim, 2005; Borchert et al., 2006). This hairpin structure is then processed by an enzyme known as Drosha, which works with the RNA-binding protein DGCR8 (DiGeorge critical region 8, called Phasa in invertebrates; Kim, 2009) to produce a ~70 nt (precursor) pre-miRNA that is then shuttled to the cytoplasm via Exportin5 and Ran-GTP61. The pre-miRNA is cleaved in the cytoplasm by an enzyme called Dicer, together with double-stranded RNA binding proteins (dsRBPs) including TRBP (Yi et al., 2003). At this stage, a double-stranded mature miRNA can be detected; however, its inhibitory capacity depends on *Dicer*-mediated incorporation of one of its strands, together with the Argonaute (Ago) protein, into the RNA-induced silencing complex (RISC; Chendrimada et al., 2005). The RISC then directs the miRNA toward a complementary “seed” sequence within the 3′ UTR of its target mRNA (Wightman et al., 1993; Reinhart et al., 2000; Pillai et al., 2005). The level of complementarity between the miRNA and its target mRNA determines whether the mRNA is degraded or translation is disrupted (Sakurai et al., 2011). Recent findings suggest that these canonical processes have many variations (Volk and Shomron, 2011). For example, insertion at 5′ UTR and coding sequences has been shown to result in similar miRNA activity, and substrates for the formation of miRNA can found be within intronic regions (mirtrons), directly spliced by RNA Pol II, thereby eluding the activity of *Drosha* (Lee et al., 2004; Ruby et al., 2007). Furthermore, they may also be derived from small nucleolar RNAs, transfer RNAs or by tRNase Z activity (Yang and Lai, 2011).

MicroRNAs are predicted to affect up to 60% of protein-coding transcripts (Lewis et al., 2005; Friedman et al., 2009), and have been implicated in essential biological processes including stem cell division particularly in mammalian development (Hatfield et al., 2005; Shcherbata et al., 2006; Qi et al., 2009), differentiation and development (Reinhart et al., 2000; Foshay and Gallicano, 2009; Xu et al., 2009; Liu et al., 2012; Zhang et al., 2012b), as well as apoptosis and cancer (Wang et al., 2008b, 2009; Kim, 2009; Corthals et al., 2011; Rissland et al., 2011; Sreekumar et al., 2011; Nie et al., 2012; Yi et al., 2012). Recent investigations using *Dicer* knockout mice have also shown that miRNA dysregulation leads to abnormalities in brain development and neuronal stem cell differentiation (De Pietri Tonelli et al., 2008; Huang et al., 2010; Kawase-Koga et al., 2010), increased cortical neurodegeneration (Davis et al., 2008; Hebert et al., 2010), and altered learning and memory (Konopka et al., 2010). Although most miRNAs are expressed in the brain, their role in neurogenesis, synaptic plasticity, and cognitive function is only beginning to be elucidated, and considerable work is required in order to better understand their influence within the context of neuropsychiatric disorders (Bredy et al., 2011; O’Connor et al., 2011). For example, among an increasing number of miRNAs implicated in schizophrenia, *miR-132* is a *CREB*-regulated miRNA driven by *NMDA* receptor signaling, which has been shown to be important for cognitive processing and is dysregulated in the brains of patients with the disorder (Kim et al., 2010; Moreau et al., 2011; Miller et al., 2012). Furthermore, *miR-219*, another *NMDA*-receptor-regulated miRNA, has been functionally linked to the behavioral impairments associated with schizophrenia (Kocerha et al., 2009). The progression of Alzheimer’s disease is characterized by cognitive decline and anxiety disorder related to dementia. Recently, Zovoilis et al. (2011) identified *miR-34c* as a negative regulator of memory consolidation in mice, with *miR-34c *also markedly increased in the brains of patients with Alzheimer’s disease. Interestingly, around the same time, it was demonstrated that the expression of *miR-34c* increases dramatically in response to acute stress, thereby providing a potential link between miRNA activity, cognitive function and anxiety in Alzheimer’s disease (Haramati et al., 2011).

More recently, we reported a role for miRNAs in regulating cognitive processes associated with fear-related anxiety disorder. Fear-extinction learning in C57/Bl6J mice led to increased expression of the brain-specific miRNA, *miR-128b*, which disrupted stability of several plasticity-related target genes and regulated formation of fear-extinction memory. We proposed that increased *miR-128b* activity might facilitate the transition from retrieval of the original fear memory toward the formation of a new fear-extinction memory, the disruption of which would have important implications for the development of fear-related anxiety disorder (Lin et al., 2011).

MicroRNA-mediated regulation of gene function has also been implicated in the development of addiction. The miRNAs *miR-124*, *Let-7d*, and *miR-181* are up-regulated in the nucleus accumbens in response to cocaine, and knockdown of each of these miRNAs influences cocaine-seeking behavior (Chandrasekar and Dreyer, 2009, 2011). Using an elegant dopamine cell type-specific knockout approach, Schaefer et al. (2010) demonstrated that *Ago2*, a subunit of Argonaute, is critical for mediating the motivational aspects of cocaine self-administration. Importantly, there are specific *Ago2*-dependent miRNAs that are cocaine-inducible, including *miR-181a*, *miR-324*, and *miR-369*, which may therefore be involved in this effect. Indeed, *miR-324* and *miR-369* regulate *MEF2* and *FosB*, two plasticity-related genes required for cognitive functioning associated with cocaine-seeking behavior (Pulipparacharuvil et al., 2008; Renthal et al., 2009). As *MEF2* has been implicated in the suppression of excitatory synaptic density (Flavell et al., 2006), a miRNA-mediated negative regulation of *MEF2* may remove this constraint and increase synaptogenesis in response to cocaine-associated learning. Furthermore, *miR-212* expression is increased in the striatum after extended access to cocaine, and over-expression of this miRNA decreases cocaine-seeking behavior (Hollander et al., 2010). Recently, it has been demonstrated that the methyl CpG-binding protein 2 (MECP2) mediates the escalation of reward-seeking behavior in rats through a homeostatic interaction with *miR-212* and subsequent regulation of brain-derived neurotrophic factor (*BDNF*) in the striatum (Im et al., 2010). This is a particularly interesting finding as it provides some of the first evidence to suggest that ncRNA-directed regulation of the epigenome occurs in an activity-dependent manner *in vivo*.

## SHORT INTERFERING RNAs

Very little is known about the role of short interfering RNAs (siRNAs) in the context of neurodegenerative disease or neuropsychiatric disorders; however, we include these small ncRNAs because of their demonstrated ability for targeting gene silencing in a variety of contexts. Similar to miRNAs, siRNAs are ~22 nt non-coding transcripts that are derived from natural antisense transcripts, pseudogenes, and repetitive sequences within endogenous dsRNA, as well as by an exogenous viral replication mechanism that is able to initiate degradation of homologous mRNAs (Sharp, 2001; Tam et al., 2008; Cech, 2011; Zhang et al., 2012c). The biogenesis of siRNAs is similar to that of miRNA; however, unlike the nuclear origin of miRNAs, they may originate in both the cytoplasm and nucleus (**Figure 1B**; reviewed in Verdel et al., 2009). This class of ncRNAs is associated with the *Ago* family as well as the *RISC* complex, which transports the guide strand to a target mRNA. Importantly, siRNAs show significantly more specific base pairing relative to miRNAs, and use *Slicer *activity to cleave and degrade their target mRNAs (Valencia-Sanchez et al., 2006). The specific base complement of exogenous siRNAs to viral mRNAs, particularly in *Drosophila* and *C. elegans*, represents an efficient antiviral immune response (Wilkins et al., 2005; Umbach and Cullen, 2009), whereas endogenous siRNAs are important for controlling somatic transposable elements in *Drosophila* (Chung et al., 2008) as well as in plants (Ito, 2011).

Perhaps the most striking quality of siRNAs is their synthetic reproducibility, which makes them attractive tools to be used in gene therapy for a variety of diseases. The first successful attempt to treat a human disease through RNAi-mediated gene silencing was demonstrated with the use of siRNA against hepatitis *C* in mice (Song et al., 2003). SiRNA has since emerged as the next “new class of drugs” (Peer and Lieberman, 2011); however, several hurdles must be overcome before siRNA-mediated gene therapy will make its way to the clinic. The primary issue is to effectively deliver siRNAs without degradation by phagocytosis or the production of off-target effects. A second concern is the design of membrane-permeable siRNAs that readily cross the blood–brain barrier (Dominska and Dykxhoorn, 2010). Advances in siRNA delivery have begun to address these challenges through the development of labeled magnetic nanoparticles, PEGylated LPD (liposome-polycation-DNA), and cationic liposomes (Li et al., 2011). siRNA has been successfully used to treat viral infections including polio, human papilloma virus, echoviruses, and adenoviruses (Morrissey et al., 2005; Saulnier et al., 2006; Tan et al., 2007; Eckstein et al., 2010). Furthermore, siRNAs have also been demonstrated to be efficacious in the treatment of human pancreatic cancer (Zhong et al., 2012), lung carcinoma (Podesta et al., 2009), and fibrodysplasia ossificans progressiva (Kaplan et al., 2011; Takahashi et al., 2011).

Reports on the putative function of both endogenous and exogenous siRNAs in the brain and their regulation of behavior are emerging. Within the context of Parkinson’s disease, a neurodegenerative disease characterized by cognitive decline, Hommel et al. (2003) found that siRNA-mediated knockdown of *Th*, which is a gene responsible for the production of tyrosine hydroxylase and a key enzyme within the dopaminergic pathway, attenuated locomotor abnormalities in a mouse model of Parkinson’s disease. Neuronal dysfunction due to mutations in the human *α-synuclein* gene affect dopaminergic levels and have been associated with idiopathic Parkinson’s disease as well as dementia with Lewy bodies. Delivery of lentiviral-mediated siRNA has been shown to effectively diminish endogenous expression of this gene both *in vitro* and *in vivo *(Sapru et al., 2006). SiRNA designed to target human *Htt*, a gene involved in Huntington’s disease, which is also a neurodegenerative disorder characterized by cognitive impairment, attenuates striatal neuropathology, and motor deficits in a mouse model of Huntington’s disease (Difiglia et al., 2007). Moreover, these studies have been extended in both mice and non-human primates, in which administration of siRNAs selective for *Huntingtin* are effective in delaying neurodegeneration, and may also have some efficacy in preventing the cognitive decline observed in Huntington’s disease (Lombardi et al., 2009; Pfister et al., 2009; Zhang et al., 2009; McBride et al., 2011; Stiles et al., 2012).

It has been widely demonstrated that exogenous siRNA is also a useful approach for inducing loss of function in studies investigating genes involved in synaptic plasticity, and learning and memory. Early research found that siRNAs could be used to silence endogenous and heterologous genes within the rat hippocampus (Krichevsky and Kosik, 2002). The important role of *GluR1* in the acquisition and consolidation of spatial memory has recently been demonstrated using siRNA (Das et al., 2012). Interestingly, knockdown of *CREB *by siRNA has elucidated the important role of *O*-GlcNAc-mediated glycolysation of *CREB* in regulating dendritic outgrowth, a process critical for the formation of long-term memory (Rexach et al., 2012). Concordantly, siRNA-mediated knockdown of the alpha 2 subunit of the *GABAA *receptor within the amygdala decreases binge drinking, thereby suggesting a potential role for endogenous siRNA in the regulation of addiction-like behavior (Liu et al., 2011). Furthermore, Panossian et al. (2012) employed an RNAi approach to demonstrate the essential role of the stress hormone neuropeptide Y (*NPY*) in regulating the stress response.

Remarkably, the stability and turnover of both miRNA and siRNA depend on the methylation status of their 3′ UTR, which potentially protects these small RNAs from urydination and exonucleolytic degradation (Ji and Chen, 2012; Zhao et al., 2012).This observation supports the prospective role of endogenous siRNAs, which is to control gene expression in mammalian oocytes (Tam et al., 2008; Watanabe et al., 2008; Nejepinska et al., 2012). These regulatory properties are also consistent with the findings of Song et al. (2011) who identified endogenous siRNAs in humans and mice, which are involved in developmental programming of male germ cells. Even more exciting is the fact that endogenous siRNAs may also direct transcriptional gene silencing in the metazoan genome through the regulation of chromatin modification. For example, in *C. elegans*,* Schizosaccharomyces pombe*, and *Drosophila* they direct H3K9 methylation to establish the formation of heterochromatin (Fagegaltier et al., 2009; Burkhart et al., 2011).

Experience-dependent expression of endogenous siRNAs has only recently been uncovered in the mammalian genome. For instance, siRNA associated with genes involved in synaptic plasticity are up-regulated in the hippocampus of mice subjected to an olfactory discrimination paradigm (Smalheiser et al., 2010). As mentioned, endogenous siRNAs are mostly derived from repetitive elements in germinal tissues, thus supporting the hypothesis that siRNAs might have evolved to sustain genomic stability during early development. However, considering the remarkable findings of Smalheiser et al. (2011), who identified an endogenous siRNA complementary to *SynGAP1*, and that dysregulation of *SynGAP1 *correlates with mental retardation as well as autism (Hamdan et al., 2009), these observations suggest a potentially important role for endogenous siRNA in cognitive disorders. Together, these early findings lay the foundation for the use of siRNA-mediated gene knockdown as a therapeutic intervention in a variety of disorders; however, it is not yet known whether manipulation of siRNAs will be applicable to the clinical treatment of cognitive deficits associated with neuropsychiatric disease.

## PIWI-INTERACTING RNAs

P-element induced wimpy testis (PIWI)-interacting RNAs (PiRNAs) are the most distinct but least investigated of all the small ncRNAs, and appear to function by repressing target gene expression within the nucleus instead of the cytoplasm (Saito et al., 2006). They are associated with a subfamily of *Ago* proteins containing an N-terminus called PAZ (from PIWI homologous Argonaute and Zwillie) and a C-terminus PIWI domain (Parker et al., 2005). PIWI proteins are highly abundant in the germline, and play an essential role in normal gonadal development in mice, with mutations resulting in male infertility (Cox et al., 1998; Girard et al., 2006; Klattenhoff and Theurkauf, 2008; Beyret and Lin, 2011). In 2001, Aravin et al. discovered a novel small ncRNA involved in the silencing of the testis-expressed *Stellate* gene in *Drosophila* but they did not realize that they were looking at PIWI protein interacting RNAs (Aravin et al., 2001; Siomi et al., 2011). Later, Watanabe et al. (2006) revealed the existence of mouse germline small RNAs between ~26 and ~30 nt long, which they named gsRNAs. Although these investigators verified an expression pattern that correlated with MIWI and MILI, two members of the PIWI protein family, they did not uncover the extent of the association and the true nature of the piRNAs. This connection was later made in mice where it was discovered that MILI and MIWI murine PIWI proteins bind, respectively, to 26 and 30 nt RNAs, which subsequently became known as piRNAs (Aravin et al., 2006; Girard et al., 2006; Grivna et al., 2006). Soon after, piRNAs were found in rat testes (Lau et al., 2006) as well as in *Drosophila* where they induced silencing of the *Suppressor of Stellate* locus found on the Y chromosome (Vagin et al., 2006).

Although it was initially suggested that piRNAs are only expressed in germinal cells (Carmell et al., 2007; Grimson et al., 2008; Beyret and Lin, 2011), they are also found in somatic tissue (Malone et al., 2009; Lee et al., 2011; Yan et al., 2011; Rajasethupathy et al., 2012). PiRNAs elude cleavage by *Dicer* and *Drosha* and are mostly derived from repetitive elements called piRNA clusters (Aravin et al., 2006; Girard et al., 2006; Grivna et al., 2006; Saito et al., 2006; Brennecke et al., 2007; Malone et al., 2009; Malone and Hannon, 2009). Although these clusters retain interspecies conservation, piRNA sequences themselves show no evolutionary conservation (Aravin et al., 2006; Girard et al., 2006; Lau et al., 2006). This interesting characteristic suggests a locus-specific function of piRNAs whereby they inhibit only those transcripts derived from the genomic region in which they originate (Lau et al., 2006). PiRNAs can also be derived from the 3′ UTR of protein-coding genes and euchromatic transposable elements (Muerdter et al., 2012), as well as from non-repetitive genomic DNA (Kim et al., 2008). In addition, through the insertion and transcription of an exogenous sequence within an endogenous piRNA cluster locus, *de novo *piRNAs can be produced from non-repetitive protein-coding sequences (Kawaoka et al., 2012). This revelation paves the way for the exploration of the role of piRNAs in the regulation of protein-coding genes through sequence-specific recognition and a *Dicer*-independent siRNA pathway, perhaps by using PIWI nuclease activity.

**FIGURE 2:**
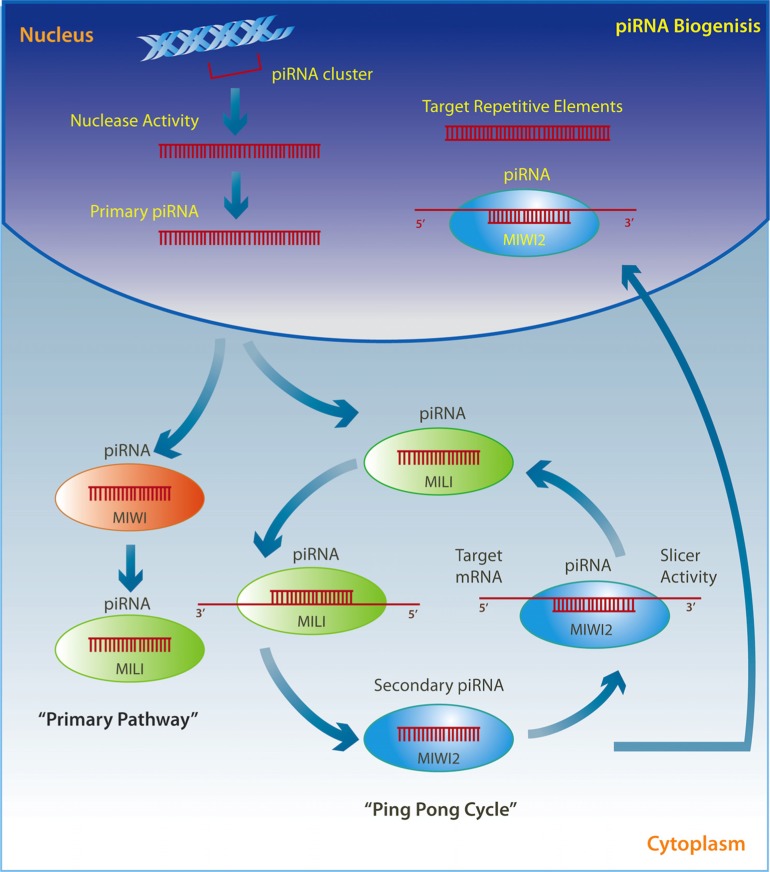
**Primary single-stranded intergenic piRNAs are transcribed within the nucleus from well-conserved clusters of repetitive elements.** Once in the cytoplasm they either interact with MIWI proteins and initiate the “primary pathway,” or directly with MILI proteins to trigger the “ping-pong cycle.” PiRNAs associated with MILI direct its slicer activity against target mRNAs, thereby producing a secondary piRNA, which is then coupled to MIWI2. MIWI2 piRNA can return to the nucleus where it targets repetitive elements, or remains within the cytoplasm where it exerts endonuclease activity on the opposite strand to reproduce a new pre-piRNA that interacts with MILI in a self-amplification loop.

PiRNAs are transcribed through two well-described mechanisms (**Figure 2**). The so called “ping pong” pathway is mainly observed in the cytoplasm of germ cells where pre-piRNAs derived from transposons are cleaved by unknown nucleases and form a complex with MILI proteins to direct MILI slicer activity against target mRNAs. This step results in a new secondary piRNA. The secondary piRNA is then coupled to a MIWI2 protein, which in turn exhibits endonuclease activity on the opposite strand and produces a new pre-piRNA, which again forms a complex with MILI in an amplification loop (Brennecke et al., 2007; Kim et al., 2009; Siomi et al., 2011; Pillai and Chuma, 2012). The MIWI2 interacting piRNA is also transported back to the nucleus where it represses transposable elements through the regulation of DNA methylation (Aravin and Bourc’his, 2008; Aravin et al., 2008; Kuramochi-Miyagawa et al., 2008; Watanabe et al., 2011). A modification may occur where pre-piRNAs are associated with MIWI proteins instead of MILI and generate a secondary piRNA that appears to remain in the cytoplasm and drives the “primary pathway,” which is present in somatic cells (Kim et al., 2009; Siomi et al., 2011; Pillai and Chuma, 2012).

Remarkably, piRNAs exploit DNA methylation and chromatin remodeling to serve their regulatory function in mammals. Mutations induced in murine MILI result in a loss of DNA methylation and a consequent up-regulation of long interspersed element-1 (LINE-1) and IAP (intracisternal A particle) elements (Aravin et al., 2007). Similarly, MIWI2 mutants also show increased expression of LINE-1 and IAP repetitive sequences, which correlate with higher DNA demethylation levels in the testes (Carmell et al., 2007). Initially, co-immunoprecipitation studies of MIWI2 failed to demonstrate a direct interaction with *DNMT3a* and *DNMT3b*, suggesting that PiRNAs do not directly regulate the activity of these enzymes (Aravin et al., 2008). However, additional investigations have shown impaired *de novo* DNA methylation and decreased piRNA levels in germline cells derived from MILI knockout mice, thus verifying a role for MILI-bound piRNAs in timing the methylation of transposons (Kuramochi-Miyagawa et al., 2008). Interestingly, Watanabe et al. (2011) proposed that piRNAs may interact with other small ncRNAs transcribed from differentially methylated genomic regions in order to drive *de novo* methylation of the locus corresponding to the associated ncRNA. PiRNAs seem to also participate in chromatin remodeling processes by recruiting major chromatin modifiers. These piRNAs, identified as piALU RNAs, have been shown to regulate chromatin organization within and around centromeres as well as by directing mechanisms of DNA repair and transcription (Blackwell et al., 2012).

Although the main role of piRNAs appears to be in the maintenance of genomic integrity and DNA stability through epigenetic silencing of transposable elements during early development (Vagin et al., 2006; Aravin et al., 2007; Brennecke et al., 2007; O’Donnell and Boeke, 2007; Dharap et al., 2011), the identification of ischemia-responsive piRNAs in the cortex of the adult rat (Dharap et al., 2011) suggests an important role for piRNAs across the lifespan. The recent demonstration that intergenic piRNAs are expressed in the mouse hippocampus, particularly within the dendritic compartment, indicates that piRNAs are functionally active in the adult central nervous system (CNS). Knockdown of piRNA *QD541777* in hippocampal neurons leads to a decreased number of dendritic spines potentially via the regulation of mRNA derived from *Cdk5rap1* and *Mark 1/2*, two genes with active roles in CNS development (Lee et al., 2011). Moreover, a cluster of four piRNAs was also found to be associated with other genes involved in dendrite formation and neuronal migration (Lee et al., 2011). Consistent with these findings, Kandel and colleagues recently discovered neuronally expressed piRNAs that promote DNA methylation of the CREB2 promoter thereby facilitating synaptic plasticity in *Aplysia *(Rajasethupathy et al., 2012). These findings suggest a potential role for piRNAs in transcriptional gene silencing and in the epigenetic facilitation of long-term memory.

Interestingly, the TUDOR protein interacts with PIWI proteins, and consequently with piRNAs, in coordinating the regulation of LINE-1 retrotransposon activity (Chen et al., 2009; Reuter et al., 2009; Siomi et al., 2010; Yabuta et al., 2011). Together with the fact that LINE-1 influences experience-dependent neuronal plasticity and somatic mosaicism within the hippocampus (Coufal et al., 2009; Muotri et al., 2009; Muotri et al., 2010), one can envision how the activity of piRNAs might be integral to these processes. It is remarkable that the current focus on the role of piRNAs is limited to the silencing of transposable elements in early development when in fact they may be potent regulators of neural plasticity and cognitive function.

## LONG ncRNAs

Long ncRNAs are transcripts over 200 nt long that can be found in both the cytoplasm and the nucleus where they exhibit tightly regulated spatiotemporal patterns of expression (Kapranov et al., 2007; Guttman et al., 2009; Khalil et al., 2009). Initially, long ncRNAs were believed to be the least conserved of all ncRNAs and to mainly serve as precursors for small RNAs (Kapranov et al., 2007). Their low level of expression also fueled the debate that they might merely be the result of transcriptional noise or artifacts of high-throughput sequencing (Struhl, 2007; Van Bakel et al., 2010). However, it has been shown that some long intergenic RNAs are under purifying selection in mammals (Ponjavic et al., 2007; Guttman et al., 2009) and exhibit cross-species conservation as well as function-specific stability (Marques and Ponting, 2009; Ulitsky et al., 2011; Clark et al., 2012). Interestingly, nuclear long ncRNAs undergo a high rate of turnover and are relatively unstable, which is concordant with the notion that they provide a mechanism for rapid response to external stimuli (Mercer et al., 2009; Clark et al., 2012). Due to these characteristics, long ncRNAs appear to be in a state of constant and rapid evolutionary development. However, short sequences nested within long ncRNAs, particularly those close to the promoter region, are highly conserved (Ponjavic et al., 2007; Guttman et al., 2010; Ulitsky et al., 2011), which suggests that they might represent core motifs for RNA binding proteins responsible for the extensive chromatin remodeling functions attributed to a variety of long ncRNAs. This would support the idea that it is the function of long ncRNAs in relation to protein-coding genes, and not their primary sequence, that is critical for their influence on gene expression and positions long ncRNAs as flexible modular scaffolds (Guttman and Rinn, 2012).

Although, the precise biogenic pathway of long ncRNAs is not yet fully understood, it involves *RNAPol II*-mediated transcription, polyadenylation, and end-capping (Guttman et al., 2009). Long ncRNAs are derived from sense and antisense strands, intergenic and intronic regions, and overlapping regions of protein-coding genes. Furthermore, novel subsets of long ncRNAs called macroRNAs are believed to serve as precursors for other small and long non-coding transcripts (Furuno et al., 2006). These might originate via *de novo* transcription of precursor non-coding transcripts, by duplication of another ncRNA or by loss of function of an ancestral protein-coding gene (Ulitsky et al., 2011). An interesting example of a long ncRNA derived from a former protein-coding sequence is *Xist*, which has arisen from the mutated protein-coding gene, *Lnx3 *(Duret et al., 2006). Moreover, *Tsx* is a long ncRNA derived from the loss of protein-coding function within the same locus (Anguera et al., 2011). It has also been proposed that long ncRNAs originate from genomic sequences from exonic regions, from chromosome rearrangement, or by duplication of the non-coding gene through retrotransposition activity and insertion of transposable elements (Ponting et al., 2009).

Long ncRNAs may function as enhancers as well as repressors through coordinated regulation of epigenetic processes, both in *cis* and in *trans*, as illustrated in **Figure 2** (Khalil et al., 2009; Orom et al., 2010a,b; Guttman et al., 2011). Considering that they account for the majority of the non-coding transcriptome (Mattick and Makunin, 2006; Jia et al., 2010), that 20% of long ncRNAs found in the human genome are bound to the polycomb group protein repressive complex (PRC), and that more than 50% of all known long ncRNAs interact with other chromatin modifying complexes such as *CoREST* and *SMCX *(Khalil et al., 2009), they are likely to be heavily involved in the epigenetic regulation of neural plasticity. Prototypical examples of epigenetic regulation directed by long ncRNAs include chromatin remodeling by *Xist*, acting in *cis*, to drive X chromosome inactivation (Duret et al., 2006), and *HOTAIR,* operating in *trans,* to silence the *HOXD* locus by recruitment of PRC2 (Rinn et al., 2007). Their role in imprinting is also evident in the activity of the paternally expressed lncRNA, *Air*, which directs silencing of *IGF2R*, *Slc22a2*, and *Slc22a3*, through the recruitment of *G9a* histone methyltransferase and methylation of H3K9 domains (Nagano et al., 2008). Another example of a long ncRNA that induces chromatin remodeling involves *Kcnq1ot1*,**which interacts with *Dnmt1* to regulate methylation of the *Kcnq1 *gene through the recruitment of the histone methyltransferases *Ezh2* and *G9a *(Mohammad et al., 2010, 2008; Pandey et al., 2008). Each of the examples further emphasizes the important role of long ncRNAs in directing epigenetic processes. Given the emerging appreciation for epigenetic mechanisms in learning and memory (Day and Sweatt, 2010), an understanding of how long ncRNAs direct the epigenome within the context of neural plasticity, cognition, and neuropsychiatric disorders is on the horizon.

Long ncRNAs have already been associated with several neurodegenerative disorders characterized by impaired cognitive function. For instance, along ncRNA isoform of the overlapping* SOX2* gene transcript called *SOX2OT* regulates CNS vertebrate development, as well as neurogenesis in adult mice (Amaral et al., 2009), and thus represents a candidate biomarker for Alzheimer’s disease (Arisi et al., 2011). Furthermore, the novel long ncRNA*17A *directs the splicing of *GPR51*, thereby producing an alternative *GABAB* receptor isoform, which results in a significant impairment in *GABAB* receptor signaling in Alzheimer’s disease (Massone et al., 2011). An additional example of the versatility of long ncRNAs in driving gene expression within the human brain is the *BACE1* antisense transcript, which prevents *miR-485-5p* induced expression of *BCAE1* gene by competing for the same binding site on its mRNA. Indeed, dysregulation of this long ncRNA has also been reported in Alzheimer’s disease (Faghihi et al., 2010). Disruption of key long ncRNAs may also play a role in the development of psychiatric disorders. For example, the long ncRNA disrupted in schizophrenia 2 (*DISC2*), is antisense to, and overlaps with, its protein-coding transcript *DISC1*. This long ncRNA has been implicated in schizophrenia, bipolar depression, and autism spectrum disorder (Millar et al., 2000; Chubb et al., 2008; Williams et al., 2009).

It is becoming clear that long ncRNAs have a direct role in the regulation of genes involved in neural plasticity and cognitive function. For example,* BDNF* supports neuronal survival and synaptic plasticity and is critically involved in learning and memory (Hall et al., 2000; Rattiner et al., 2004a,b; Bredy et al., 2007). A human antisense transcript called *BDNFOS* or *antiBDNF* was previously shown to interact with its sense mRNA, *BDNF *(Pruunsild et al., 2007). Interestingly, very recently a conserved antisense transcript to *BDNF *has been discovered in mice, which inhibits *BDNF *transcription by recruitment of EZH2, a key component of the epigenetic silencing complex, PRC2. Additionally, administration of a sequence-specific inhibitor called an antagoNAT or siRNA-mediated knockdown of *BDNF*-AS both lead toup-regulation of *BDNF* mRNA and protein expression *in vitro* and *in vivo*, resulting in increased neurite outgrowth and maturation (Modarresi et al., 2012).

The potential importance of long ncRNA activity related to higher order cognitive processing is also highlighted by the discovery of human accelerated region 1 (*HAR1F*), which is expressed in Cajal–Retzius neurons and was one of the first long ncRNAs shown to be involved in human neocortical development (Pollard et al., 2006). Recently it was found that repression of *HAR1F* by *REST* leads to its decreased expression in the striatum of Huntington’s disease (Johnson et al., 2010), and that *REST* also interacts with *DGCR5* and *TUG1 *lncRNAs leading to DiGeorge syndrome and Huntington’s disease (Johnson et al., 2009; Johnson, 2011). Johnson (2012) extended these investigations and confirmed that both *TUG1* and *NEAT1* are significantly up-regulated in Huntington’s disease brains, whereas *MEG3* and *DGCR5 *showed decreased expression.

**FIGURE 3:**
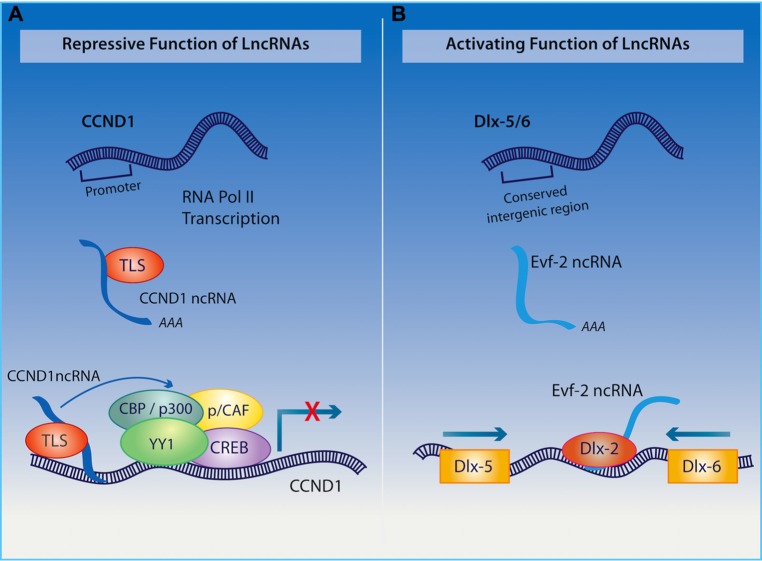
**(A)** Upon receiving a signal for DNA damage, the long ncRNA CCND1 binds to the TLS protein to direct a protein complex to the CCND1 target gene where allosteric modification of CREB and the histone acetyltranserases (HATs) CBP and p300 are produced, which results in gene silencing. **(B)** Long ncRNA Evf2 is transcribed from an ultra conserved intergenic region within the Dlx-5/6 locus. Binding of this long ncRNA to the Dlx-2 protein triggers transcriptional activation of the Dlx-5/6 enhancer region in a homeodomain-specific manner.

Among other functions, the organization of the nuclear architecture also seems to require the transcription of the polyadenylated long ncRNA *NEAT1*, which is associated with nuclear paraspeckles (Clemson et al., 2009; Souquere et al., 2010), and *NEAT2* (also known as *MALAT1 *– metastasis-associated lung adenocarcinoma transcript 1), which regulates synapse formation (Bernard et al., 2010). Furthermore, the long ncRNA Cycling D1 (*CCND1*) interacting with a protein called translocated in liposarcoma (*TLS*; as indicated in **Figure 3)**, acts as a transcriptional co-repressor and as a sensor of DNA damage by binding and repressing both *CREB* and *p300 *acetyltransferase leading to the silencing of their target gene (Wang et al., 2008a). Given the important role of *CREB* and *p300* in regulating neural plasticity and cognitive function, it would be interesting to determine whether a disruption of the long ncRNA *CCND1* can cause impairments in cognitive function similar to those observed when either *CREB* or *p300 *is inhibited.

The ability of long ncRNAs to interact with transcription factors also endows them with a remarkable capacity to orchestrate mammalian neurogenesis, which will undoubtedly have an influence on cognition. For example, the long non-coding transcript* Evf2, *derived from the *Dlx5/6* gene locus, binds to the homeodomain of *Dlx-2* forming an enhancer-like complex that activates *Dlx5/6* expression (as described in **Figure 3**; Feng et al., 2006). The relevance of this long ncRNA in the hippocampus and cognition is exemplified through studies on *Evf2* knockout mice, which exhibit impaired synaptic plasticity and spatial memory as a result of aberrant interneuron development (Bond et al., 2009). Extending these discoveries, Ng et al. (2012) have demonstrated that long ncRNAs interact with transcription factor *SOX2* as well as PRC2, with the authors suggesting that they are required for pluripotency of neural progenitor cells. Additionally, long ncRNAs have been shown to direct that activity of other ncRNAs within the CNS. Indeed, the cytoplasmic long ncRNA, *lncRNA_N2*, harboring miRNA *miR-125B*, regulates the transcription of this small RNA and indirectly promotes neurogenesis.

More excitingly, long ncRNAs have also been associated with drug addiction and, potentially, fear-related anxiety disorder. For instance, the long ncRNAs *MIAT*, *MEG3*, and *NEAT1/2* are up-regulated within the nucleus accumbens of heroin abusers (Michelhaugh et al., 2011). Accordingly, a previous genome-wide association study has implicated *MEG3* with potential predisposition to heroin addiction (Nielsen et al., 2008). Interestingly, *MIAT* is associated with the nuclear matrix and is involved in oligodendrocyte development (Mercer et al., 2010), whereas *MEG3* is a cAMP-responsive, maternally imprinted, long ncRNA that is expressed in distinct subpopulations of neurons (Zhao et al., 2006; Michelhaugh et al., 2011) and contributes to early neurogenesis (Mercer et al., 2010). More recently, the lncRNA *RMST*, also known as dorsomedial telencephalon gene 2 (*DMT2*), was revealed to interact with nuclear and chromatin factors during the development of dopaminergic neurons (Ng et al., 2012). Additionally, the long ncRNA *BC1* has been shown to direct the activity of the dopamine 2 receptor (*Drd2*) as well as metabotropic glutamate receptors (*mGluR*), which are required for balancing neuronal excitability at the synapse (Zhong et al., 2009), and that *BC1*^-^^/^^-^mice develop increased anxiety (Centonze et al., 2007). Considering the important role of *Drd2* and *mGluRs* in regulating the extinction of conditioned fear, it is possible that a dysregulation of the long ncRNA *BC1* contributes to the development of fear related anxiety disorders.

Through their interaction with various epigenetic regulatory proteins, long ncRNAs might serve as molecular brakes on transcriptional activity, as originally proposed by McQuown and Wood (2011) within the context of HDAC activity and memory formation, which in our opinion would also contribute greatly to rapid, experience-dependent, variations in genomic function. Consequently, it is anticipated that long ncRNAs will have profound effects on neural plasticity and cognition associated with neuropsychiatric disorders. In summary, cell type-specific patterns of long ncRNA expression may serve to coordinate the fidelity of synaptic plasticity and neural connectivity by dynamically monitoring and integrating multiple transcriptional and post-transcriptional events (Mercer et al., 2008; Qureshi and Mehler, 2011).

Finally, as described in **Table 1**, there are also many other classes of ncRNAs not discussed in this review, the understanding of which is only starting to emerge.

**Table 1:** Identification and known characteristics of other ncRNAs.

NcRNA	Size	Characteristics	Reference
eRNAs	~100 bp to 91 kb	Show homologous chromatin modifications to protein coding genes, aid in enhancement of non-specific cell neighboring genes expression. A subtype identified in mouse neuronal cells shows association with enhancers and positive correlation with their levels of mRNA synthesis	Kim et al. (2010), Orom et al. (2010a,b)
TERRAs	100 bp to 9 kb	Conserved in mammals localized in the nucleoplasm in all mammalian chromosomes at telomeric regions of interphase and metaphase cells. RNA Pol II and developmental stage Dependant. Potentially involved in eukaryotic heterochromatin conformation, telomere length and function in human iPS cells and regulation of telomerase activity	Azzalin et al. (2007), Schoeftner and Blasco (2008), Deng et al. (2009), Luke and Lingner (2009), Redon et al. (2010), Yehezkel et al. (2011)
tel-sRNAs	~24 bp	Evolutionary conserved, potential involvement in telomere structure and function,suggested to be regulated by epigenetic mechanisms	Cao et al. (2009)
TASRs	20–200 bp	Clustering at the 3′ end. Conserved in mouse and human genomes with yet undescribed functions but potential role in gene expression suggested	Kapranov et al. (2007), Carninci et al. (2008), Zhang et al. (2012a)
aTASRs	Unspecified	5′ poly U short RNAs antisense to 3′ ends of annotated regions undetermined functionality	Kapranov et al. (2010)
PASRs	26–50 bp	Mapped within 500 nt of knownTSS. Mostly overlapping with 5′ end of protein coding genes. Association with RNA Pol II, Histone H3 and H4 acetylation and potential up regulation of neighboring genes	Kapranov et al. (2007), Carninci et al. (2008), ENCODE Transcriptome Project (2009)
PALRs	200 bp to 1 kb	Overlapping with 5′ end of protein coding genes. Potential up regulation of protein coding genes showing evolutionary conserved sequences	Kapranov et al. (2007)
PROMPTs	~200-600 bp	Transcribed from upstream regions of annotated TSSs, they are polyadenylated, highly unstable and mostly restricted to the nucleus. Suggested to affect promoter methylation and regulate transcriptional processes	Preker et al. (2008, 2011)
TiRNAs	~15 to 30 bp	Enriched in the nucleus, transcribed from downstream of TSS sequences are linked to CpG rich promoters, transcription factor binding, and widespread transcription initiation inducing up regulation of protein coding genes	Taft et al. (2009, 2010)
Stress-tiRNAs	Unspecified	5′ and 3′ end small-derived RNAs from tRNAs are induced to inhibit translation as a stress response pathway	Emara et al. (2010)
spliRNAs	~18 bp	Developmental stage and region specific expression, Dicer and Drosha independent pathways.Weakly expressed, associated with highly expressed loci	(Taft et al., 2010)
moRNAs	~20 bp	Processed by nuclear Drosha, 5′ derived transcripts from of miRNAs precursors, weakly expressed and with unknown functions	Langenberger et al. (2009), Taft et al. (2010)

Although the biogenesis of most of these non-coding transcripts remains unclear, their tissue-specificity and developmental stage-dependent expression provides strong evidence for their biological relevance justifying the need for further research into the complex regulatory potential hidden within the eukaryotic transcriptome.

## OUTLOOK AND CONCLUSION

The huge repertoire of ncRNAs, that we are only beginning to recognize, suggests a sophisticated RNA-mediated layer of control over genomic function that has evolved to coordinate tissue- and developmental stage-specific regulation of gene expression. The redundancy in consensus sequences typical of most ncRNAs allows for a highly efficient transcriptional regulation of protein-coding genes, whereas the potential stability provided by the methylation status of most small ncRNAs supports their well established role in regulating gene expression. In the case of some long ncRNAs, their low level of conservation may contribute directly to their function by enabling the cell to respond to external stimuli with greater flexibility, thereby conferring a rapidly adaptive plasticity that is distinct from, and more complex than, the much slower acting protein-coding genes (Mattick, 2011). Consequently and given that ncRNAs are highly expressed in the brain, it is anticipated that they will play a significant role in driving neural connectivity within learning and behavioral adaptation of higher eukaryotes. This firmly positions ncRNAs at the forefront of cell-to-cell communication within the context of cognitive function and potentially in the development of neuropsychiatric disorders. Given that a significant proportion of known long ncRNAs interact with repressive complexes, future investigations will expand on these observations through the application of innovative techniques including high-throughput sequencing together with UV-crosslinking and immunoprecipitation (HITS-CLIP; Zhang and Darnell, 2011), chromatin isolation by RNA purification (ChIRP; Chu et al., 2011), capture hybridization analysis of RNA combined with RNA sequencing (CHART; Simon et al., 2011), and RNA-capture-Seq (Mercer et al., 2011). Such studies will aid in the determination of novel ncRNAs and their genome-wide binding sites, which will be important to elucidate the essential role of RNA-directed epigenetic regulation of gene function. Most studies on ncRNAs have been based on stem cell and embryonic development, with the function of miRNAs in the adult brain only recently becoming appreciated. Moreover, *in vitro* studies examining long ncRNAs and piRNAs, and their influence on neural plasticity, are emerging. It will be exciting to see how these *in vitro *findings translate *in vivo *and to elucidate whether the biological significance of these ncRNAs will extend to memory and cognition. Furthermore, RNAi will be a useful tool to facilitate the knockdown of ncRNAs that are associated to neuropsychiatric disorders, such as *TUG1* and *NEAT1*. Consequently, if lncRNAs truly represent modular scaffolds, then it will be imperative to determine the stability of the epigenetic processes directed by all classes of ncRNAs, particularly those resulting from rapidly changing behavioral responses. Given the fact that endogenous siRNAs are mostly represented in oocytes, whereas piRNAs are strongly expressed in the male germ line, each capable of exerting their own genomic function, also makes them attractive candidates to investigate their potential influence on genomic imprinting and transgenerational epigenetic inheritance. Finally, since many ncRNAs remain transcriptionally quiescent until a specific developmental stage is met or an appropriate environmental signal is received (Kretz et al., 2012), it will be important to decipher the mechanisms coordinating such precise and rapid genomic responsiveness. The central dogma of protein-coding genes controlling the eukaryotic genome represents an archaic constraint on our journey to truly expand the understanding of gene–epigenome–environmental interactions. It is undeniable that this layer of gene regulation has many fundamental biological and functional roles yet to be explained and it might be within neural plasticity, cognition, and neuropsychiatric disorders where the lasting signature of ncRNAs could be at their most significant.

## Conflict of Interest Statement

The authors declare that the research was conducted in the absence of any commercial or financial relationships that could be construed as a potential conflict of interest.
